# FluxMap: A VANTED add-on for the visual exploration of flux distributions in biological networks

**DOI:** 10.1186/1752-0509-6-33

**Published:** 2012-05-01

**Authors:** Hendrik Rohn, Anja Hartmann, Astrid Junker, Björn H Junker, Falk Schreiber

**Affiliations:** 1 Leibniz Institute of Plant Genetics and Crop Plant Research (IPK), Molecular Genetics, Corrensstr. 3, Gatersleben, 06466, Germany; 2 Leibniz Institute of Plant Genetics and Crop Plant Research (IPK), Physiology and Cell Biology, Corrensstr. 3, Gatersleben, 06466, Germany; 3 Institute of Computer Science, Martin Luther University Halle-Wittenberg, Von-Seckendorff-Platz 1, Halle, 06120, Germany

## Abstract

**Background:**

The quantification of metabolic fluxes is gaining increasing importance in the analysis of the metabolic behavior of biological systems such as organisms, tissues or cells. Various methodologies (wetlab or drylab) result in sets of fluxes which require an appropriate visualization for interpretation by scientists. The visualization of flux distributions is a necessary prerequisite for intuitive flux data exploration in the context of metabolic networks.

**Results:**

We present FluxMap, a tool for the advanced visualization and exploration of flux data in the context of metabolic networks. The template-based flux data import assigns flux values and optional quality parameters (e. g. the confidence interval) to biochemical reactions. It supports the discrimination between mass and substance fluxes, such as C- or N-fluxes. After import, flux data mapping and network-based visualization allow the interactive exploration of the dataset. Various visualization options enable the user to adapt layout and network representation according to individual purposes.

**Conclusions:**

The Vanted add-on FluxMap comprises a comprehensive set of functionalities for visualization and advanced visual exploration of flux distributions in biological networks. It is available as a Java open source tool from http://www.vanted.org/fluxmap.

## Background

Metabolic flux analysis is becoming increasingly important for the understanding of the dynamics of metabolic networks [1]. Flux values can be derived from wetlab experiments, e. g. using stable isotopic tracers (such as ^13^ C-Metabolic Flux Analysis), or from drylab computational approaches, such as Flux Balance Analysis (see, for example, [2].) Based on comparative flux analysis conclusions can be drawn about the influence of internal or external parameters on the metabolic behavior of a biological system. The visualization of fluxes in the context of metabolic networks supports such analysis. Firstly, images are a convenient way of communicating information to the scientist and, secondly, visual analysis of network-associated fluxes is intuitive for the user. Thirdly, especially for flux distributions in large networks, flux visualization enables data interpretation on a global level and facilitates the gain of knowledge. As an extension to network-based flux visualization, interactive exploration techniques provide the basis for an easy and fast comparison of different flux distributions, which would be very elaborate when working with raw data in tabular form.

An adequate tool for the visual exploration of flux distributions has to provide the following functionalities: (1) user-friendly input of external flux data as biochemical reaction formulas, including experimental specifications such as time points, different species and substance weights to represent C- or N-fluxes, (2) import of biological networks from different databases, (3) network editing capabilities, (4) validation of the input data set, (5) network-based visualization of flux values and visualization options for individual user requirements, (6) visualization of quality parameters, (7) interactive comparison of different flux maps, e. g. from different species and (8) output of flux visualization in different (graphical) formats.

**Figure 1:**
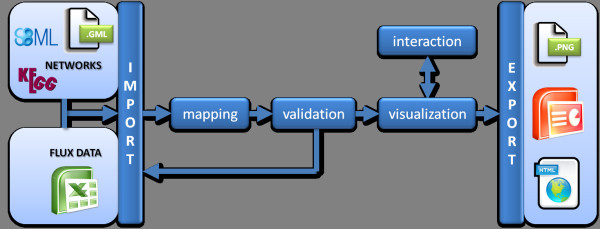
**Schematic workflow of FluxMap.** FluxMap requires the import of structured flux data and metabolic networks (SBML, KGML or GML). The flux measurement values are mapped to the metabolic network and validation can be performed. In case of invalid flux distributions, the imported data should be re-checked. The visualization of a valid flux distribution can be adapted to individual purposes and exported in several file formats. Various interaction options enable the visual and comparative exploration of flux distributions in the context of biologicalnetworks.

Here we present FluxMap, a Vanted [3] add-on specialized on the visualization and exploration of flux distributions, supporting all above mentioned requirements. The FluxMap workflow schema depicted in Figure 1 and an example shown in detail in Additional file 1.

## Implementation

### Data input

**Figure 2:**
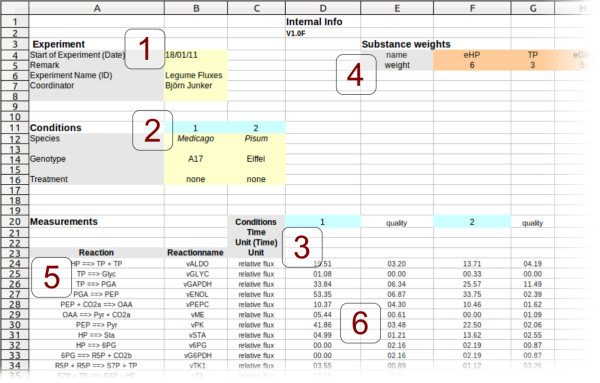
**Data input template.** Flux data input template specifying: (1) experimental metadata, (2) the conditions, (3) the time points/samples, (4) the substance weights, e. g. number of C-atoms, (5) the reaction-equations and (6) the flux values including quality information. The template shows data from the first use case described in the Results section.

For the import of flux data, FluxMap makes use of a structured Excel template file (see Figure 2). In fields 1 and 2 the user is asked to specify experimental metadata such as experiment annotation and information about different conditions. Field 3 contains information about time points which have been considered in the course of the experiment. In case of the visualization of substance fluxes, the user needs to specify the substance weights (assigning atom numbers to metabolites) in field 4. For visualization of mass fluxes the atom number has to be set to 1 for each metabolite. Reaction formulas have to be defined in field 5 with the corresponding flux measurement values in field 6. The reaction formula consists of two arrow-separated lists of reactant and product substances. Stoichiometric factors are part of the reaction formula and are allowed to be non-integer numbers. Furthermore, the input template supports the assignment of quality parameters (e. g. confidence interval) to each flux measurement value. Taken together the template enables users to import and save the flux measurement data together with experiment metadata in a well-structured way.

### Network representation and mapping

In the process of parsing the template file, FluxMap generates a dataset which is adapted to the hypergraph properties of metabolic networks. Considering the stoichiometry of the reactant, the flux measurement value of the reaction and the substance weights, reactions are represented as bipartite graphs by adding a reaction node (labeled with the reaction name) connecting all metabolites participating in this reaction with edges to or from this node. The parsed dataset then can be mapped to a biochemical network. These networks can be drawn manually in Vanted or imported in various file formats (SBML, KGML, GML, SIF) from the local file system or external pathway sources such as KEGG [4] and MetaCrop [5]. During the mapping process the flux values are automatically extracted from the parsed template and represented as edge thickness between the corresponding nodes. The direction of edges in the network will be adapted according to the flux data. Zero fluxes are indicated by a dashed line, thereby visually preserving the topology of the network even for sparse flux data. In case of reactions in the template which are not represented in the network, FluxMap enables the user to automatically add corresponding network elements.

### Balance validation

**Figure 3:**
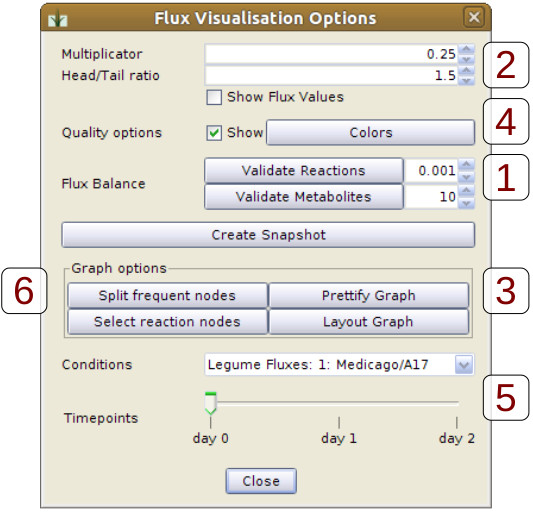
**Visualization and interaction options of FluxMap.** This dialog offers various functionalities for visualization and interactive exploration of flux distributions such as: (1) validation of reaction and metabolite balance (2) changing global edge thickness and ratio of arrowhead/-tail, (3) improved visualization by rounding reaction nodes, (4) visualization of quality information using color graduation, (5) interactive exploration of flux distributions using a combobox and a slider and (6) splitting of frequently occurring nodes (e. g. ATP and CO_2_).

The data mapping step is followed by a validation step for checking the flux balance of each reaction (see dialog in Figure 3, field 1). For balanced flux distributions, the sum of all ingoing fluxes into a reaction node is equal to the sum of all outgoing fluxes. If this condition is not fulfilled, the reaction will be reported to the user as unbalanced. Several reasons might account for a reaction being unbalanced. In most cases this is due to typographical errors in the input template, such as misspelled substance names (“Sucr” instead of “Suc”), wrong stoichiometry (“2Suc” instead of “2 Suc”), missing stoichiometry and missing reactions or reactants. Another reason is given by the nature of the flux the user wants to analyze. In mass flux maps, which describe the flux of molecules per time unit, certain reactions cannot be balanced (all reactions with different numbers of substrates and products). Therefore balance validation is only applicable for fluxes of atom numbers per time unit, e. g. computed carbon flux distributions or data from ^13^ C labeling experiments.

The validation step supports users in tracking down typographical errors in the input template which facilitates the import and analysis of large and complex flux data sets.

### Visualization

**Figure 4:**
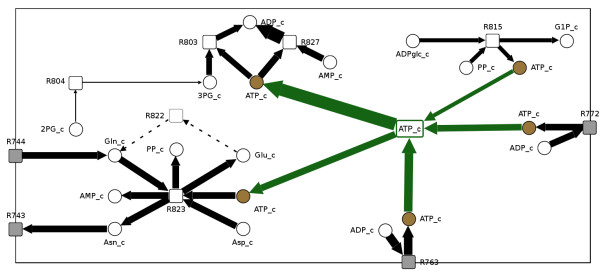
**Interaction.** Part of a flux map, in which the nodes of the widely distributed substance “ATP_c” (brown nodes) were temporarily and interactively connected (green node and edges) in order to support tracking of main flux directions in large maps. Gray nodes indicate transport reactions to or from other compartments.

**Figure 5:**
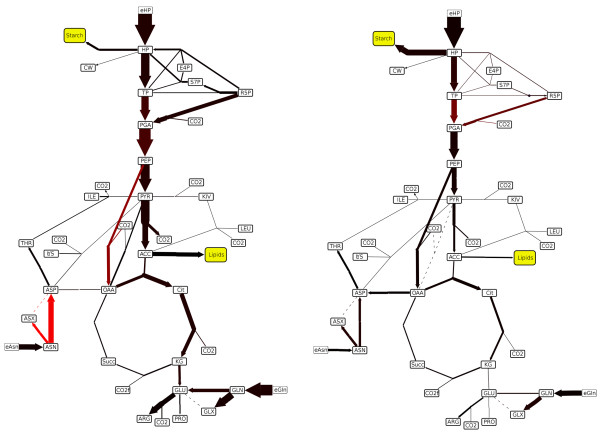
**13C fluxes in legume seeds.** Network of central metabolism with ^13^C measurements from seeds of Medicago truncatula (left, lipid producing seed) and Pisum sativum (right, starch producing seed). Red graduation indicates the measurement confidence (red = low, black = high) and reaction nodes obtained a rounded appearance. Yellow nodes indicate the main seed products starch and lipids.

FluxMap offers a simple dialog which can be used to parameterize the visualization of the flux distribution according to individual needs (see Figure 3). The multiplicator parameter adapts the global edge thickness of all fluxes (Figure 3, field 2). The arrowhead/-tail ratio might be varied in order to emphasize the edge direction rather than the edge thickness (field 2). Different reaction node visualization styles can be chosen, e. g. invisible, small, rounded or normal nodes with or without labels (field 3). It is additionally possible to visualize the quality parameters using a configurable color graduation, thereby highlighting potential measurement errors (field 4; compare also Figures 4 and 5, red saturation). Each of these parameter modifications triggers an instant redraw of t he flux map. Instead of the representation of flux values as edge thickness, flux data might also be visualized using charts, e. g. bar- or line-charts which are assigned to edges or the reaction nodes. General network editing functionalities (node and edge coloring, node size, node shape) are implemented in Vanted and accessible through FluxMap.

### Interaction

As specified in the input template, the flux dataset can comprise values representing different samples and varying conditions (Figure 2, fields 2 and 3). This complexity in flux data sets creates the need for the interactive exploration by switching between these experimental factors. In order to meet this requirement, FluxMap offers a combobox (for all conditions) and a slider (for all time points), enabling users to explore and visually compare corresponding flux maps (Figure 3, field 5).

**Figure 6:**
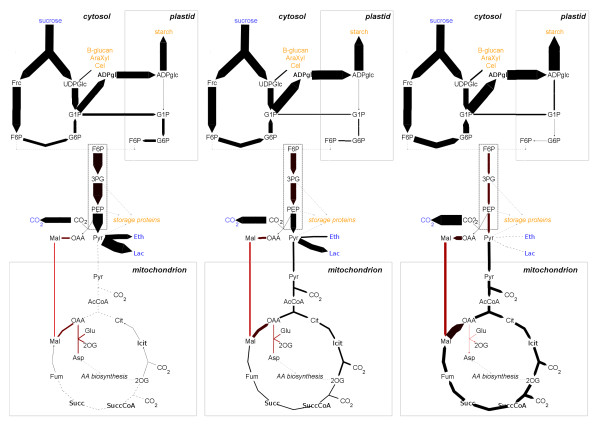
**Flux balance analysis in barley seed metabolism.** Mass flux maps depicting fluxes within central barley seed metabolism during the anoxic phase (left), the hypoxic phase (middle) and the aerobic phase (right). Metabolites taken up or excreted by the model are highlighted in blue. Metabolites incorporated into biomass are highlighted in orange. Red graduation indicates high flux variability. Note, that mass flux maps are not balanced.

Network layout provides the basis for network visualization and exploration. Especially in large networks it is indispensable to follow the fluxes throughout the whole map. FluxMap supports several automatic layouting algorithms such as the DOT-layout and additionally offers flux-specific layouts including the functionality for splitting nodes with a high degree of connectivity. Usually these nodes represent substances taking part in many different biochemical reactions, such as ATP or CO_2_. The user may select such nodes based on a node list sorted by degree of interconnection, and thus trigger an automatic splitting of these nodes into a number of cloned nodes, one for each reaction (see Figure 3, field 6). Node splitting on the one hand improves network layout but on the other hand makes it difficult to visually follow the global flux. Therefore, FluxMap enables the user to temporarily re-connect nodes with the same label again (see green fluxes in Figure 6). This interaction can be repeated successively for several metabolites, thereby facilitating the tracking of main flux directions throughout large maps.

### Data output

The (flux enriched) network can be saved in the GML or GraphML file format. The graphical export is possible as raster images (PNG, JPG) and vector images (SVG, PDF and PowerPoint). Using the noncommercial tool ImageJ [6], raster images may be used to create animated GIF files, e. g. in order to be able to show animations between different flux distributions in PowerPoint slides. Furthermore, FluxMap supports export as clickable web pages.

## Results

### ^13^C measurement of metabolic fluxes in seeds

In order to evaluate carbon flux distributions of the central seed metabolism of *Pisum sativum and Medicago truncatula*^13^C measurements were carried out based on the experimental procedure from Junker et. al. [7] and raw data was processed according to Wiechert et. al. [8]. From a biological point of view the comparison of both plants is of interest, as on the one hand they belong to the same family, indicating a high genetic similarity, but on the other hand show a strong physiological difference: the Pisum sativum seed primary product is starch, whereas the Medicago truncatula seed primary product are lipids (compare Figure 4). To understand the differences of the metabolism from a systemic perspective, both flux maps have to be visualized and compared interactively. The data is taken directly from the output of 13CFLUX, which is a toolkit for isotope-based metabolic flux analysis [8]. The dataset consists of two conditions, a list of how many carbon atoms the substances have, the relative flux values and a quality value indicating measurement uncertainty. Using the combobox in FluxMap, users are able to compare both flux maps, including the quality of the fluxes. By switching between both conditions it is easy to see that for Medicago truncatula there is a lower flux into starch, but a higher flux into lipids in comparison to Pisum sativum (yellow nodes). Further statistical evaluation may be carried out to search for other significant differences or correlations.

### Flux balance analysis of oxygen conditions in barley seed metabolism

Grafahrend-Belau et. al. [9] generated a stoichiometric model of barley seed central metabolism and analyzed the impact of six different oxygen levels on the rate of biomass synthesis (biomass is composed of all metabo- lites highlighted orange in Figure 4). Using flux balance analysis they derived simulated flux distributions for different oxygenic phases (interpreted as conditions). The visualization focuses on mass fluxes rather than substance fluxes and, consequently, the weights of all metabolites were set to 1 in the input template. Additionally flux variability analysis (FVA) was performed, which gives the minimal and maximal possible flux through a reaction, when observing all possible flux distributions that fulfill the given constraints (for further explanations see [10]). FVA is used for the detectionmof essential reactions or reactions allowing for am high flux variation, thereby providing robustness against environmental influences. In the present use casemthis variability information is assigned to each reaction as a quality parameter. If the “quality” is zero, them flux through this reaction does not vary and is drawn black (Figure 4). More variable reactions have “high quality” values and are visualized by red graduation. FluxMap enables users to easily switch between the different oxygen conditions while displaying the quality information simultaneously. The simulated flux distributions for three different oxygen regimes (see Figure 4) are in accordance with published experimental results (see [9] for a detailed discussion): under anaerobic conditions a high flux through fermentative enzymes is predicted whereas aerobic conditions lead to a higher flux into storage products and seed biomass. Furthermore, different oxygen levels affect the TCA cycle in terms of cyclic or non-cyclic modes and the direction of its reactions. Note that FluxMap automatically adapts the edge directions.

## Discussion

FluxMap provides functionalities for the advanced visualization and exploration of flux maps, regardless of source and type of the flux data. Following the argumentation of Sweetlove and Ratcliffe [11], who conclude that flux distributions derived from Flux Balance Analysis provide an important complement to experimentally determined ^13^ C-flux distributions, FluxMap supports the visual analysis of mass and substance fluxes.

For the visualization of flux measurement data other software exists with partially overlapping features compared to FluxMap, as summarized in Table 1. The majority of tools provide the possibility to import flux data from other sources. This is not true for MetaFluxNet and OptFlux, which compute and analyze internal simulation-derived flux data. On the other hand, only these tools and FluxMap are able to process substance and mass fluxes. As a common feature, most of the tools offer direct access to network databases as well as network editing functionalities, which are important prerequisites for network-based flux visualization. Once flux data is mapped onto a network only the FluxMap workflow comprises a flux balance validation step. With regard to visualization and exploration functionalities, it is comparable to tools such as FluxViz, fa-BINA and Omix with options for customization and interaction. In the context of advanced visualizations, FluxMap supports the representation of quality parameters such as the confidence interval for each flux value. Finally most of the tools allow for the integration of other -omics data in order to set fluxomics in relation to metabolomics, transcriptomics as well as proteomics.

**Table 1:** Tool comparison

	FluxViz [13]	fa-BINA [14]	Omix [15]	BioCyc Omics Viewer [16]	Reactome Skypainter [17]	Pathway Projector [18]	MetaFluxNet [19]	OptFlux [20]	FluxMap
import									
external flux data	+	+	-	+	+	+	-	-	+
biochemical reactions	-	+	+	-	-	-	+	+	+
substance/mass fluxes	-	-	-	-	-	-	+	+	+
access to network databases	+	+	+	+	-	-	+		+
network editing	+	+	+	-	-	+	+	+	+
balance validation	-	-	-	-	-	-	-	-	+
visualization									
customization	+	+	+	-	-	-	-	-	+
confidence/quality	-	-	+	-	-	-	+	+	+
exploration	+	+	+	-	-	-	-	-	+
other omics data	+	+	+	+	+	+	-	-	+
flux computation	-	+	+	-	-	-	+	+	-

Feature overview of tools available for flux visualization in the context of networks. “+” indicates good support of the feature, “-” indicates an absent or inadequately supported feature. In conclusion, FluxMap supports a broad range of features and novel functionality for the visual exploration of flux distributions in biological networks.

In summary, FluxMap combines a simple, fast and source-independent data import of flux data with different parameters for generating user-specific visualizations as well as intuitive network exploration capabilities. FluxMap and the underlying Vanted system provide a framework for the integration of comprehensive metabolic datasets and their combined representation in the network context. Other add-ons of the Vanted system enable FluxMap to e. g. access experimental and pathway databases and add support for the Systems Biology Graphical Notation [12].

## Conclusions

FluxMap is a tool for the visualization and interactive exploration of flux distributions in biological net- works and available from Additional file 2. Flux data import is achieved using a structured template containing experimental metadata as well as flux values and quality parameters each of which is assigned to one biochemical reaction. After the import and mapping of the flux data, balance validation can be used for the identification of possible input errors. Various visualization and interaction options enable users to explore, compare between and interact with visualized flux maps of different conditions and samples. As an extension of the Vanted system, FluxMap also provides network editing functionalities and further options for data analysis such as statistical tests. In conclusion, the FluxMap tool fulfills all requirements of flux data visualization and, together with basic Vanted functionalities, constitutes a comprehensive software package for advanced, network-based analysis of flux and other metabolic datasets.

### Availability and requirements

· Project Name: FluxMap

· Project home page: http://www.vanted.org/fluxmap/

· Operating system(s): Platform independent (Java)

· Programming language: Java 6/7

· License: GPL 2.0

## Authors’ contributions

HR implemented the tool. HR, AH and AJ wrote the manuscript. BHJ and AH provided requirements. BHJ and AH evaluated the implementation. FS supervised the project and gave conceptual advice. All authors read and approved the manuscript.

## Competing interests

The authors declare that they have no competing interests.

## Supplementary Material

Additional file 1 Workflow screenshots - tutorial.pdf. A PDF containing a series of screenshots of an exemplary FluxMap workflow. Click here for file

Additional file 2 Software and source code - FluxMap.jar. The Java binary and the source code as a JAR-file, which can be installed and used as an add-on for Vanted. Click here for file
