# Supplementary material for: FluxMap: A VANTED add-on for the visual exploration of flux distributions in biological networks
Source: BMC Syst Biol. 2012 May 1;6:33. doi: 10.1186/1752-0509-6-33 (PMC3403919; doi:10.1186/1752-0509-6-33)
Supplement: Additional file 1 — Workflow screenshots - tutorial.pdf. A PDF containing a series of screenshots of an exemplary FluxMap workflow. [file 1752-0509-6-33-S1.pdf]

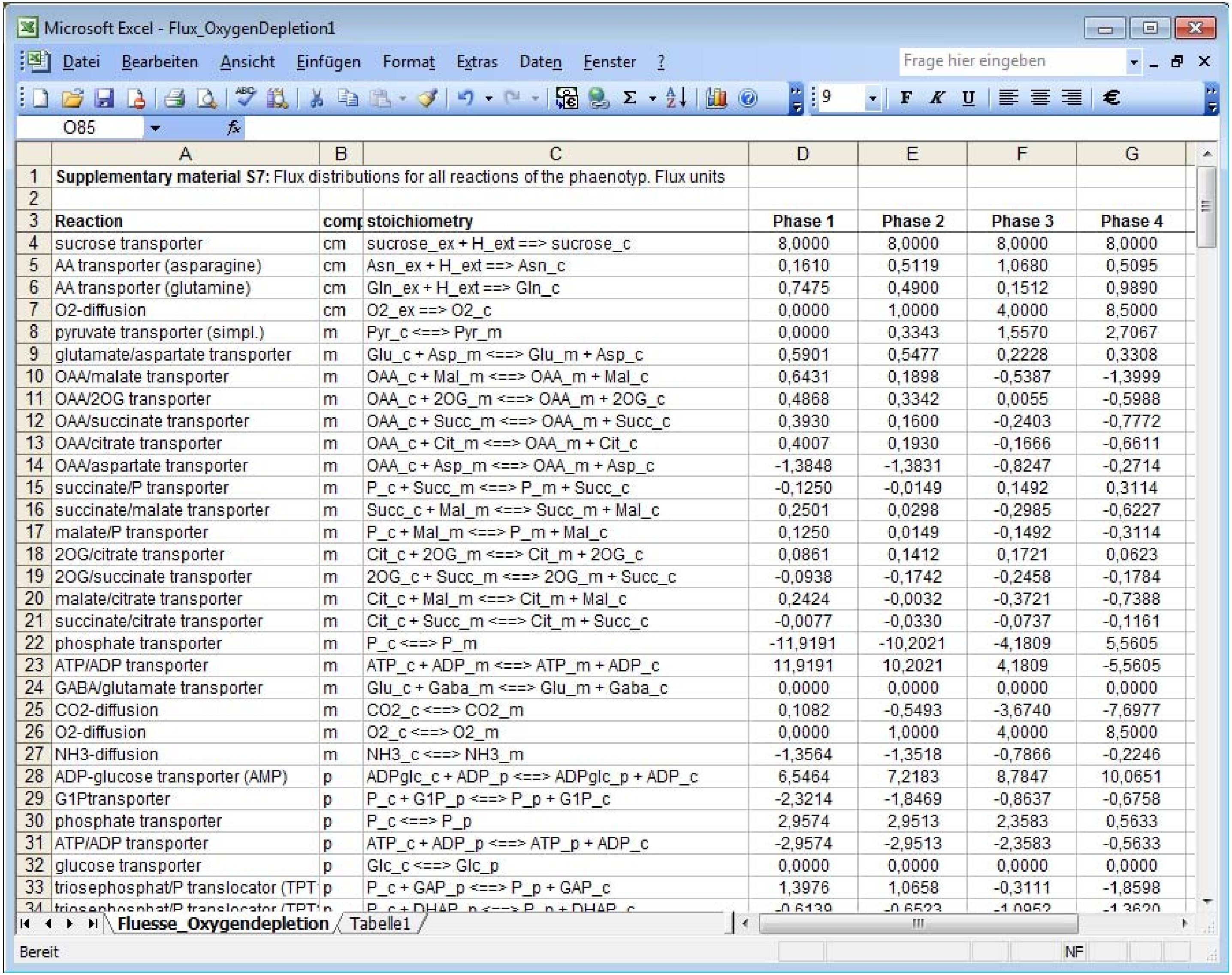

	A	B	C	D	E	F	G
1	**Supplementary material S7:** Flux distributions for all reactions of the phaenotyp. Flux units						
2							
3	**Reaction**	**comp**	**stoichiometry**	**Phase 1**	**Phase 2**	**Phase 3**	**Phase 4**
4	sucrose transporter	cm	sucrose_ex + H_ext ==> sucrose_c	8,0000	8,0000	8,0000	8,0000
5	AA transporter (asparagine)	cm	Asn_ex + H_ext ==> Asn_c	0,1610	0,5119	1,0680	0,5095
6	AA transporter (glutamine)	cm	Gln_ex + H_ext ==> Gln_c	0,7475	0,4900	0,1512	0,9890
7	O2-diffusion	cm	O2_ex ==> O2_c	0,0000	1,0000	4,0000	8,5000
8	pyruvate transporter (simpl.)	m	Pyr_c <==> Pyr_m	0,0000	0,3343	1,5570	2,7067
9	glutamate/aspartate transporter	m	Glu_c + Asp_m <==> Glu_m + Asp_c	0,5901	0,5477	0,2228	0,3308
10	OAA/malate transporter	m	OAA_c + Mal_m <==> OAA_m + Mal_c	0,6431	0,1898	-0,5387	-1,3999
11	OAA/2OG transporter	m	OAA_c + 2OG_m <==> OAA_m + 2OG_c	0,4868	0,3342	0,0055	-0,5988
12	OAA/succinate transporter	m	OAA_c + Succ_m <==> OAA_m + Succ_c	0,3930	0,1600	-0,2403	-0,7772
13	OAA/citrate transporter	m	OAA_c + Cit_m <==> OAA_m + Cit_c	0,4007	0,1930	-0,1666	-0,6611
14	OAA/aspartate transporter	m	OAA_c + Asp_m <==> OAA_m + Asp_c	-1,3848	-1,3831	-0,8247	-0,2714
15	succinate/P transporter	m	P_c + Succ_m <==> P_m + Succ_c	-0,1250	-0,0149	0,1492	0,3114
16	succinate/malate transporter	m	Succ_c + Mal_m <==> Succ_m + Mal_c	0,2501	0,0298	-0,2985	-0,6227
17	malate/P transporter	m	P_c + Mal_m <==> P_m + Mal_c	0,1250	0,0149	-0,1492	-0,3114
18	2OG/citrate transporter	m	Cit_c + 2OG_m <==> Cit_m + 2OG_c	0,0861	0,1412	0,1721	0,0623
19	2OG/succinate transporter	m	2OG_c + Succ_m <==> 2OG_m + Succ_c	-0,0938	-0,1742	-0,2458	-0,1784
20	malate/citrate transporter	m	Cit_c + Mal_m <==> Cit_m + Mal_c	0,2424	-0,0032	-0,3721	-0,7388
21	succinate/citrate transporter	m	Cit_c + Succ_m <==> Cit_m + Succ_c	-0,0077	-0,0330	-0,0737	-0,1161
22	phosphate transporter	m	P_c <==> P_m	-11,9191	-10,2021	-4,1809	5,5605
23	ATP/ADP transporter	m	ATP_c + ADP_m <==> ATP_m + ADP_c	11,9191	10,2021	4,1809	-5,5605
24	GABA/glutamate transporter	m	Glu_c + Gaba_m <==> Glu_m + Gaba_c	0,0000	0,0000	0,0000	0,0000
25	CO2-diffusion	m	CO2_c <==> CO2_m	0,1082	-0,5493	-3,6740	-7,6977
26	O2-diffusion	m	O2_c <==> O2_m	0,0000	1,0000	4,0000	8,5000
27	NH3-diffusion	m	NH3_c <==> NH3_m	-1,3564	-1,3518	-0,7866	-0,2246
28	ADP-glucose transporter (AMP)	p	ADPglc_c + ADP_p <==> ADPglc_p + ADP_c	6,5464	7,2183	8,7847	10,0651
29	G1Ptransporter	p	P_c + G1P_p <==> P_p + G1P_c	-2,3214	-1,8469	-0,8637	-0,6758
30	phosphate transporter	p	P_c <==> P_p	2,9574	2,9513	2,3583	0,5633
31	ATP/ADP transporter	p	ATP_c + ADP_p <==> ATP_p + ADP_c	-2,9574	-2,9513	-2,3583	-0,5633
32	glucose transporter	p	Glc_c <==> Glc_p	0,0000	0,0000	0,0000	0,0000
33	triosephosphat/P translocator (TPT	p	P_c + GAP_p <==> P_p + GAP_c	1,3976	1,0658	-0,3111	-1,8598
34	triosephosphat/P translocator (TPT	p	P_c + DHAP_p <==> P_p + DHAP_c	-0,6139	-0,6523	-1,0952	-1,3620

Figure 1: Take flux raw data

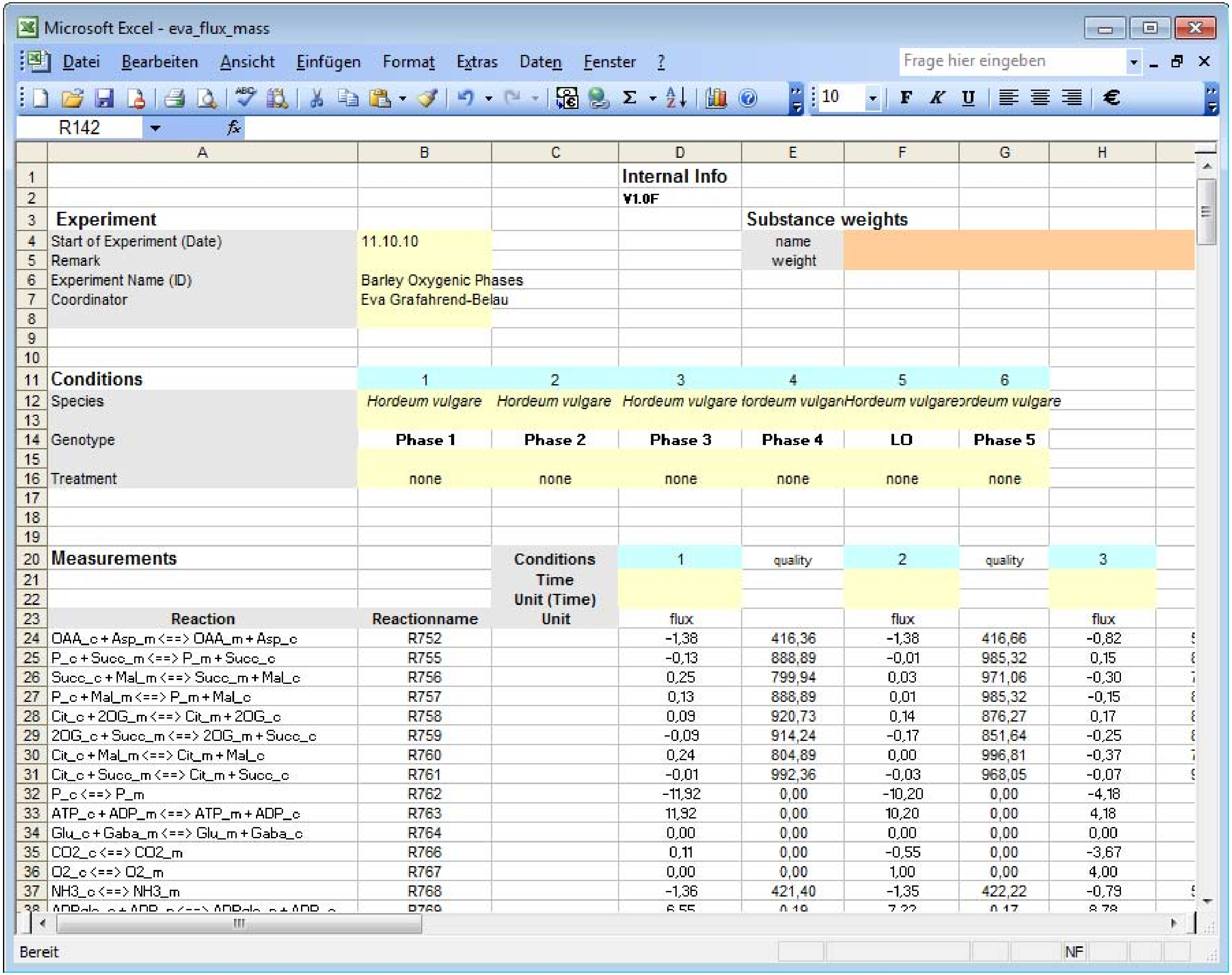

Microsoft Excel - eva_flux_mass

	A	B	C	D	E	F	G	H
1				Internal Info				
2				V1.0F				
3	Experiment				Substance weights			
4	Start of Experiment (Date)	11.10.10			name			
5	Remark				weight			
6	Experiment Name (ID)	Barley Oxygenic Phases						
7	Coordinator	Eva Grafahrend-Belau						
8								
9								
10								
11	Conditions	1	2	3	4	5	6	
12	Species	*Hordeum vulgare*	*Hordeum vulgare*	*Hordeum vulgare*	*lordeum vulgar*	*Hordeum vulgare*	*ordeum vulgare*	
13								
14	Genotype	**Phase 1**	**Phase 2**	**Phase 3**	**Phase 4**	**LO**	**Phase 5**	
15								
16	Treatment	none	none	none	none	none	none	
17								
18								
19								
20	Measurements		Conditions	1	quality	2	quality	3
21			Time					
22			Unit (Time)					
23	Reaction	Reactionname	Unit	flux		flux		flux
24	OAA_c + Asp_m <==> OAA_m + Asp_c	R752		-1,38	416,36	-1,38	416,66	-0,82
25	P_c + Succ_m <==> P_m + Succ_c	R755		-0,13	888,89	-0,01	985,32	0,15
26	Succ_c + Mal_m <==> Succ_m + Mal_c	R756		0,25	799,94	0,03	971,06	-0,30
27	P_c + Mal_m <==> P_m + Mal_c	R757		0,13	888,89	0,01	985,32	-0,15
28	Cit_c + 2OG_m <==> Cit_m + 2OG_c	R758		0,09	920,73	0,14	876,27	0,17
29	2OG_c + Succ_m <==> 2OG_m + Succ_c	R759		-0,09	914,24	-0,17	851,64	-0,25
30	Cit_c + Mal_m <==> Cit_m + Mal_c	R760		0,24	804,89	0,00	996,81	-0,37
31	Cit_c + Succ_m <==> Cit_m + Succ_c	R761		-0,01	992,36	-0,03	968,05	-0,07
32	P_c <==> P_m	R762		-11,92	0,00	-10,20	0,00	-4,18
33	ATP_c + ADP_m <==> ATP_m + ADP_c	R763		11,92	0,00	10,20	0,00	4,18
34	Glu_c + Gaba_m <==> Glu_m + Gaba_c	R764		0,00	0,00	0,00	0,00	0,00
35	CO2_c <==> CO2_m	R766		0,11	0,00	-0,55	0,00	-3,67
36	O2_c <==> O2_m	R767		0,00	0,00	1,00	0,00	4,00
37	NH3_c <==> NH3_m	R768		-1,36	421,40	-1,35	422,22	-0,79
38	[illegible]	R769		[illegible]	[illegible]	[illegible]	[illegible]	[illegible]

Bereit

Figure 2: Fill data input template

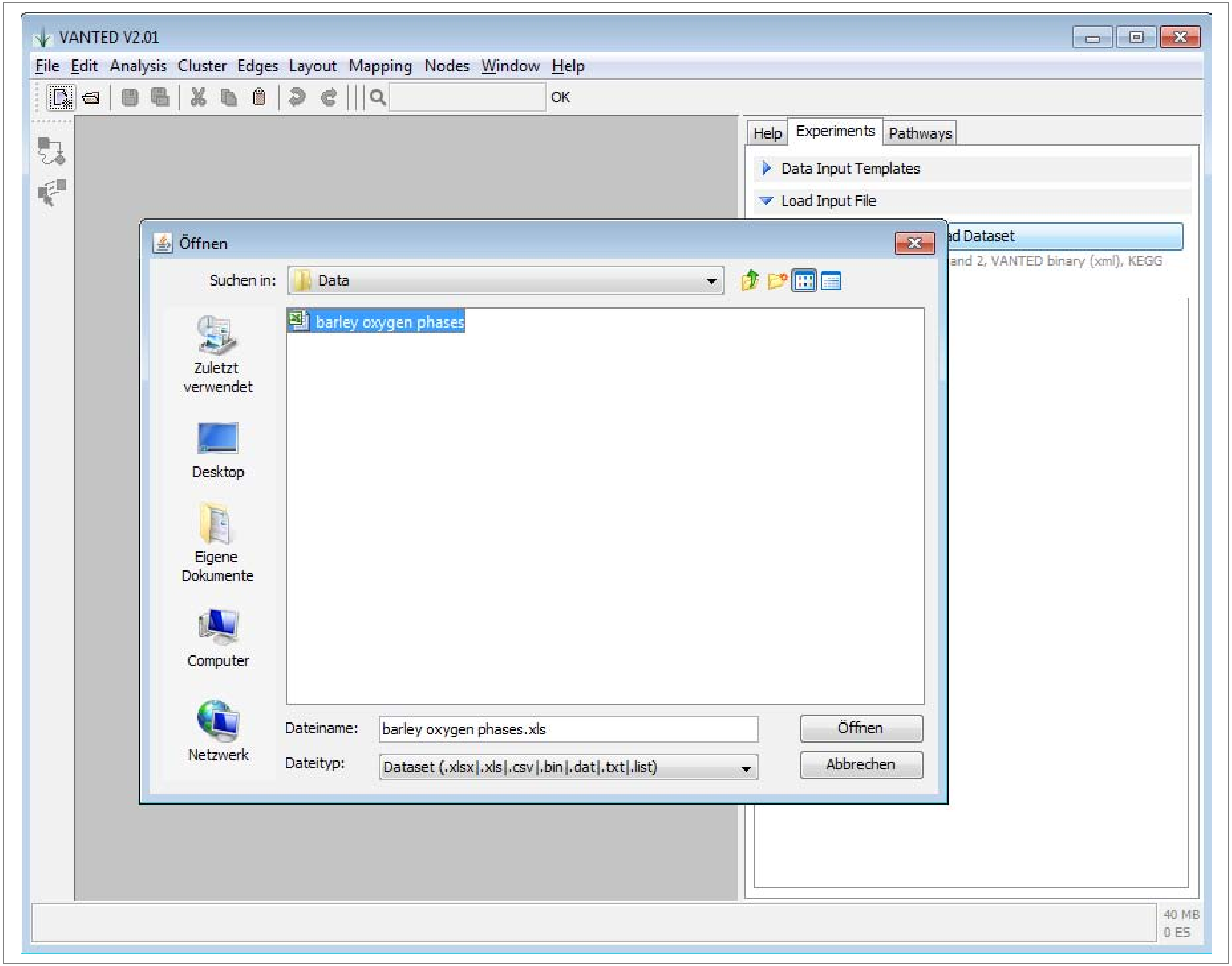

Figure 3: Load flux data

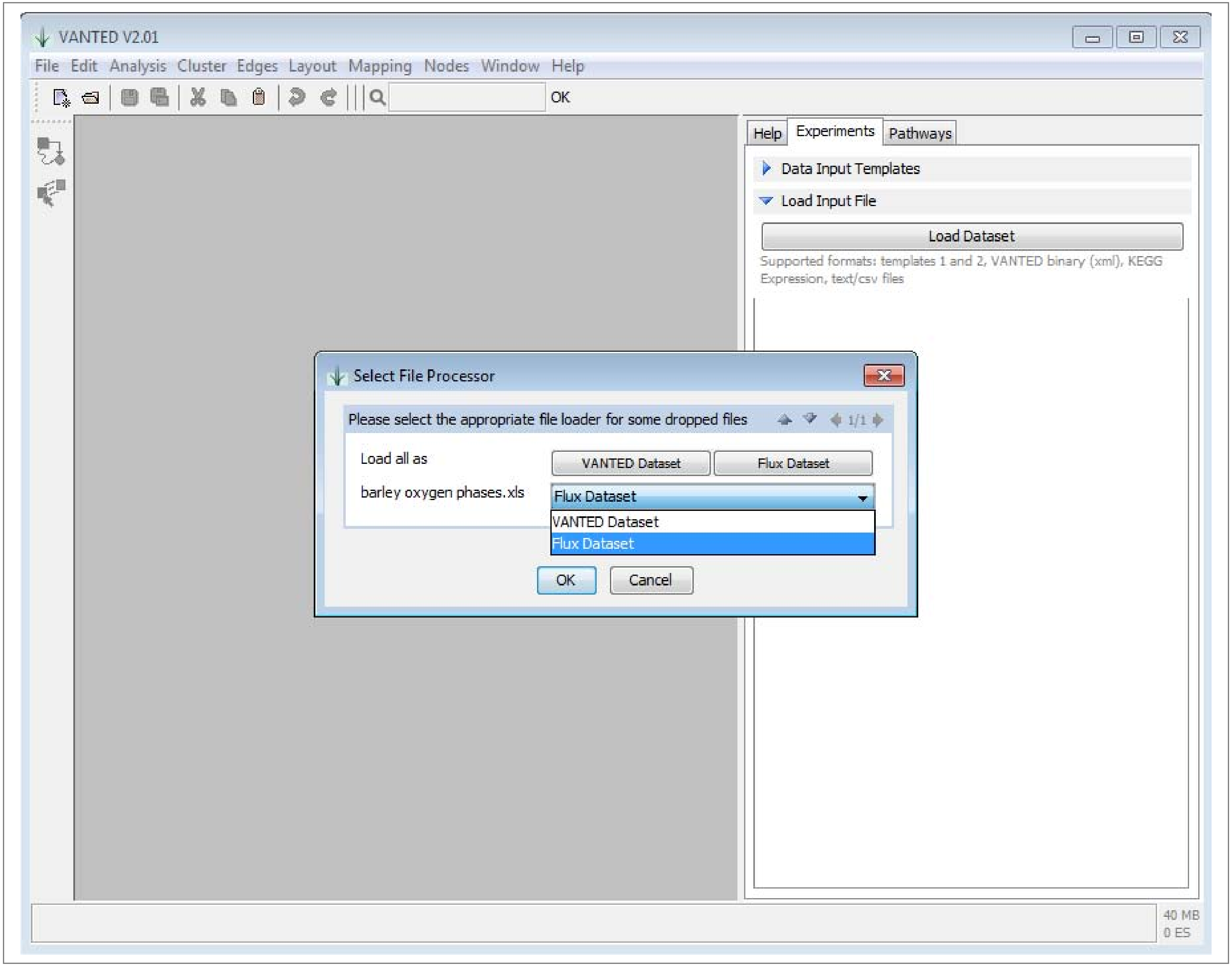

Figure 4: Select correct template loader

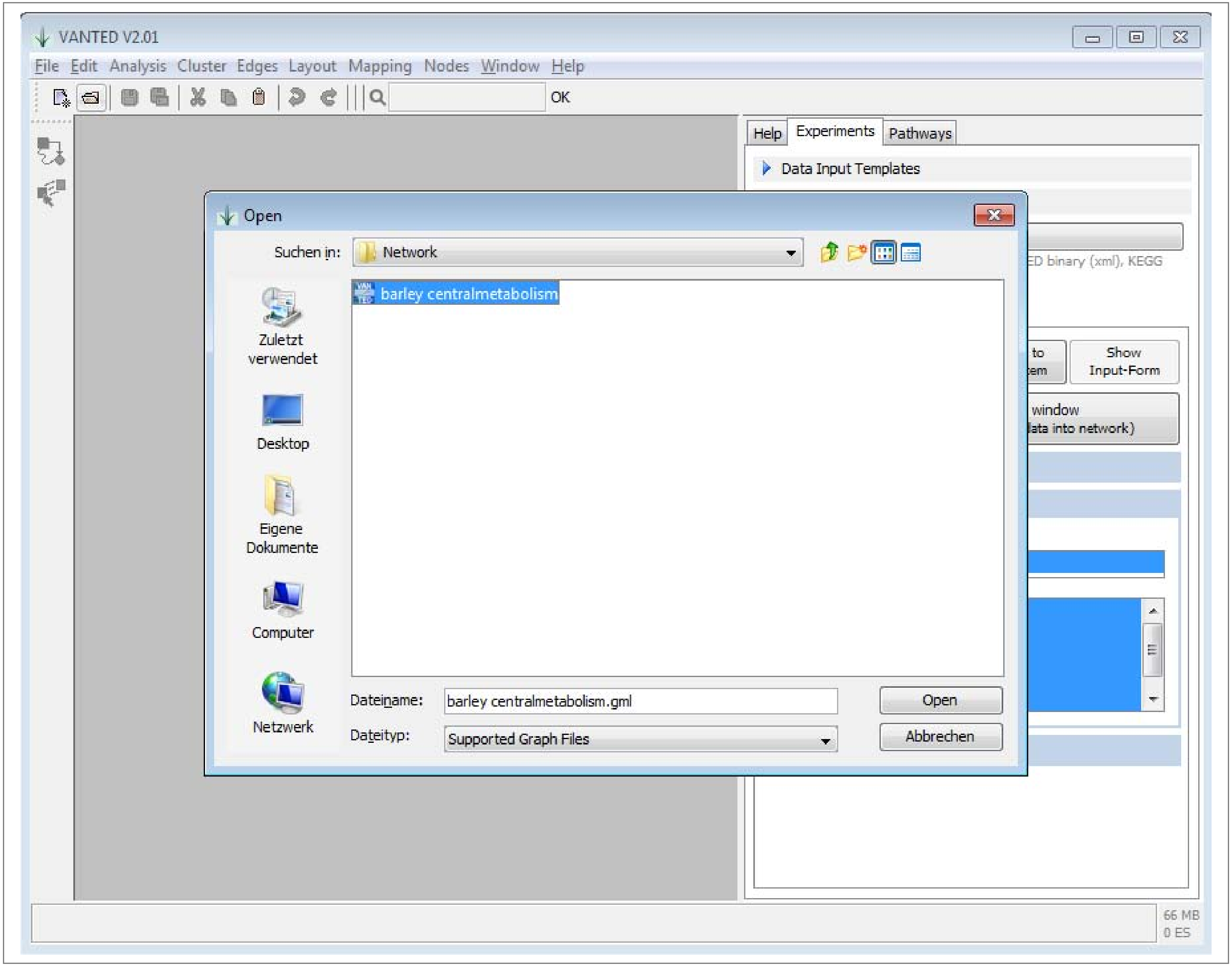

Figure 5: Load network

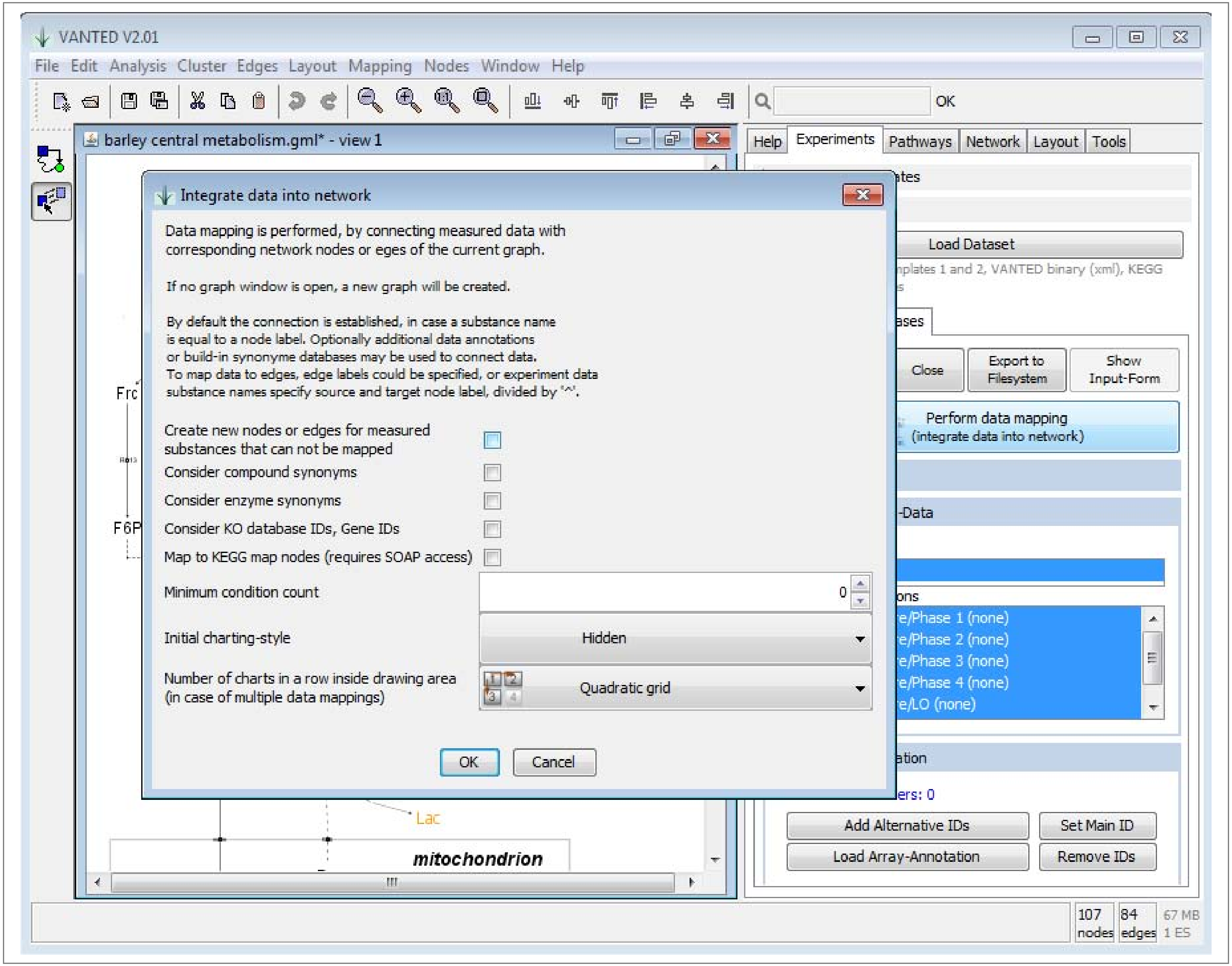

Figure 6: Map flux data onto network

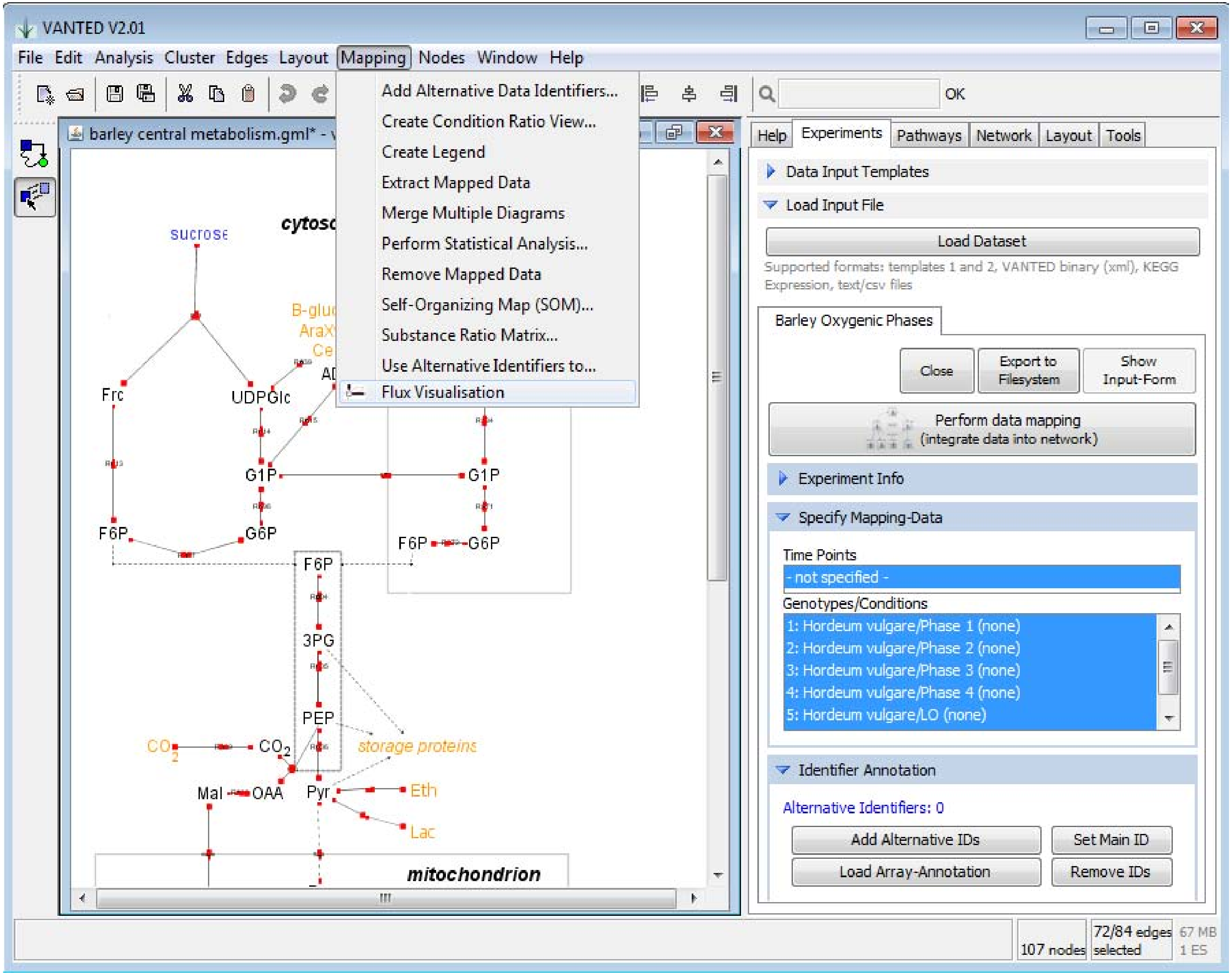

Figure 7: Summon FluxMap dialog

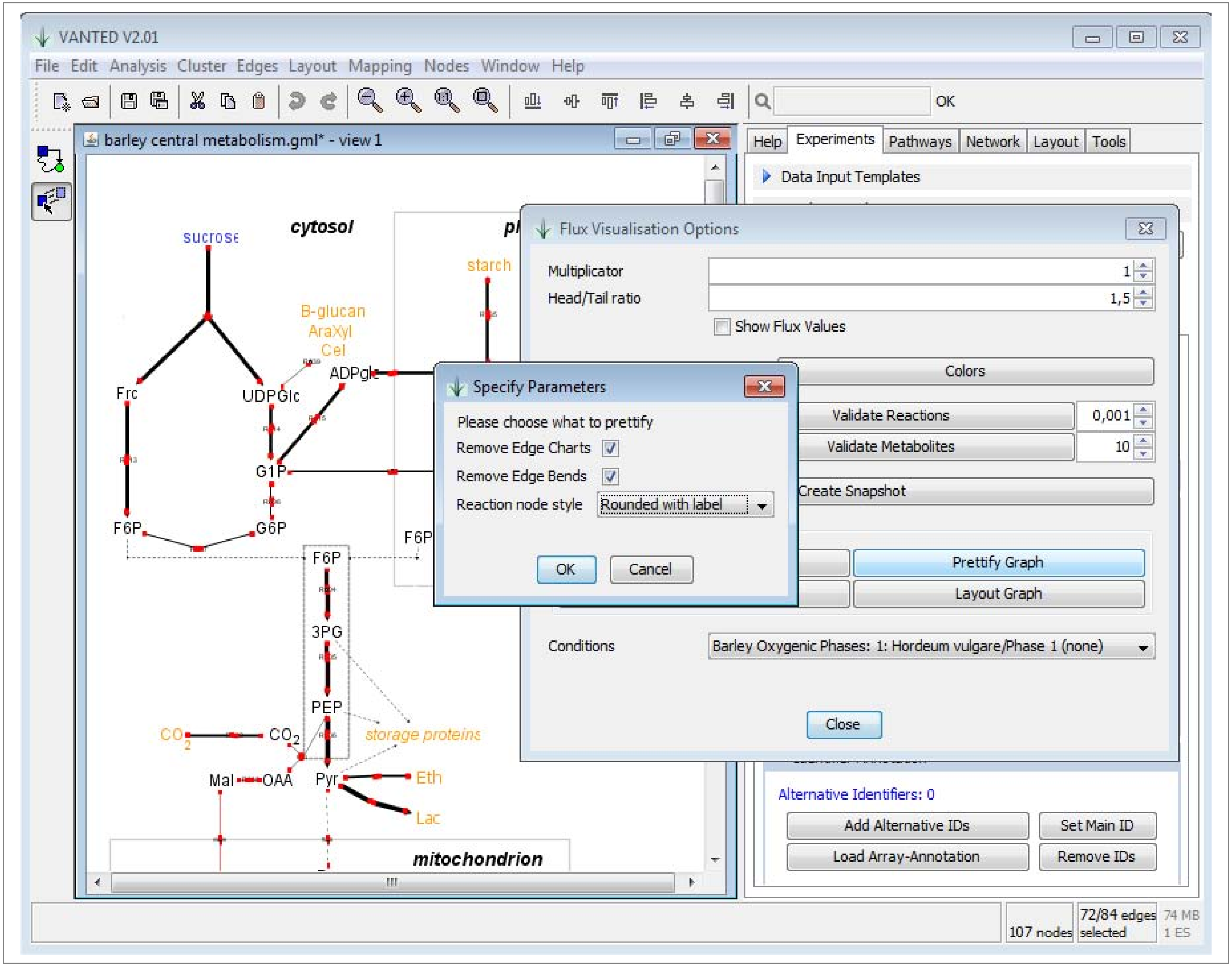

Figure 8: Improve visualization

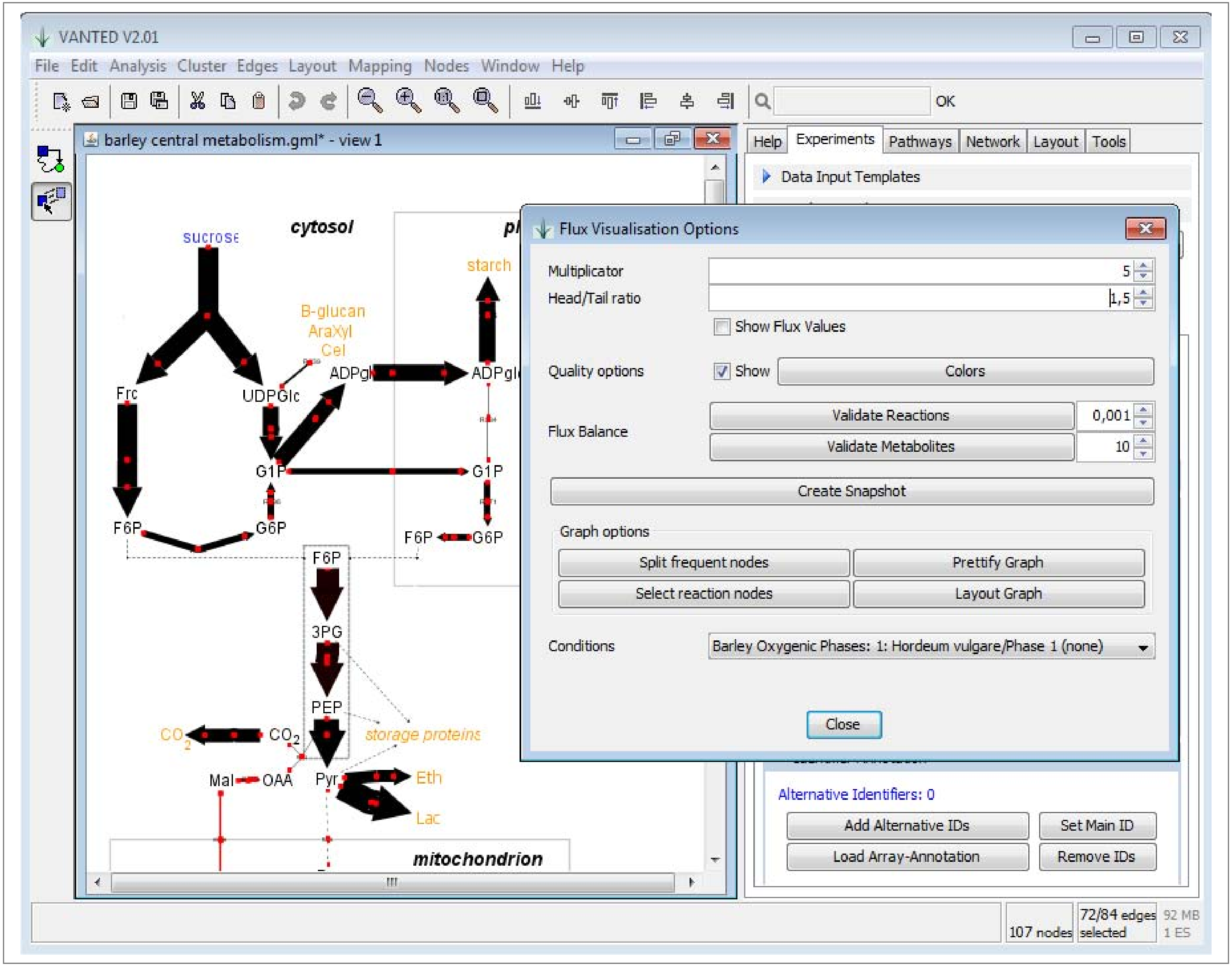

Figure 9: Increase global edge thickness

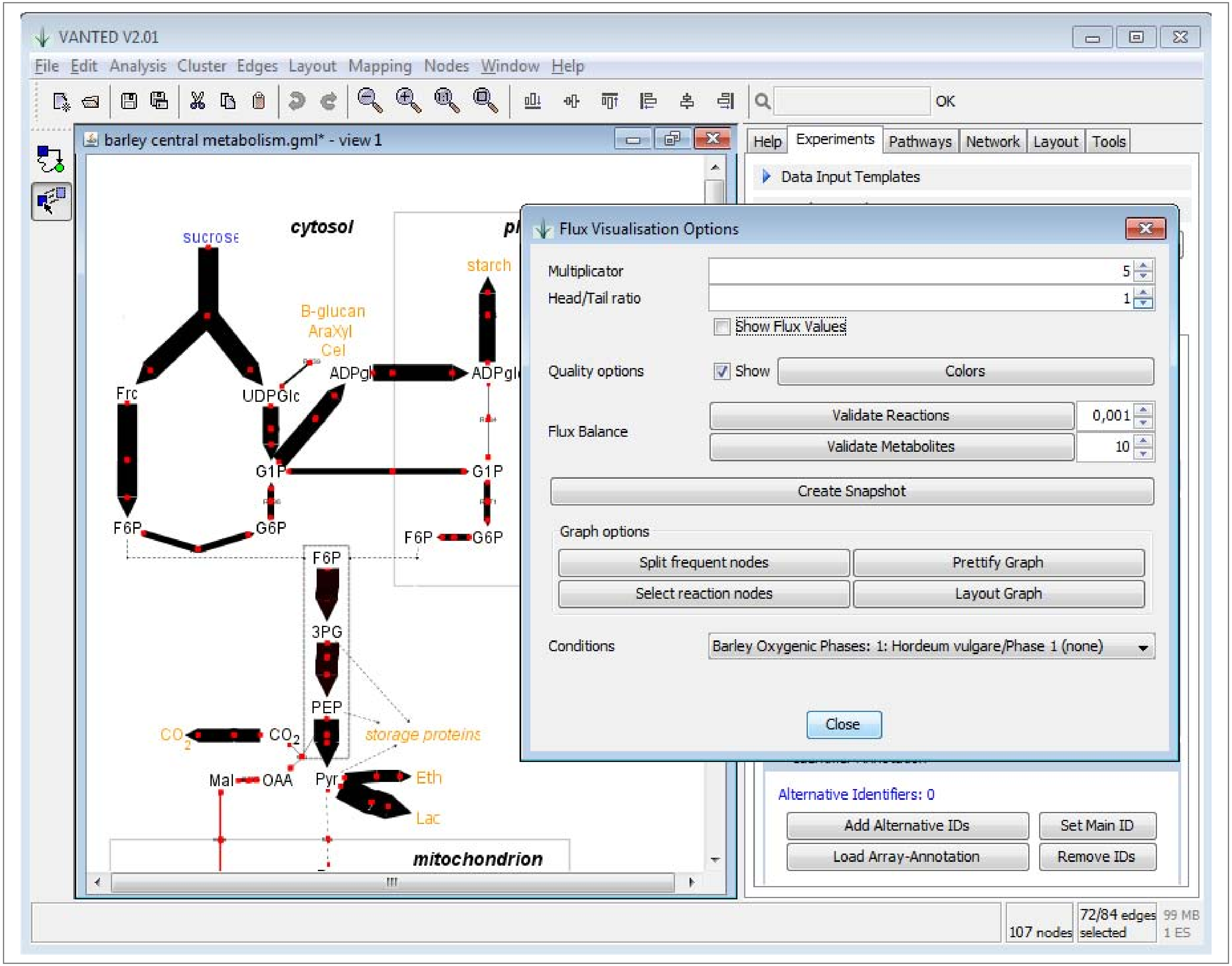

Figure 10: Reduce arrowhead/-tail ratio

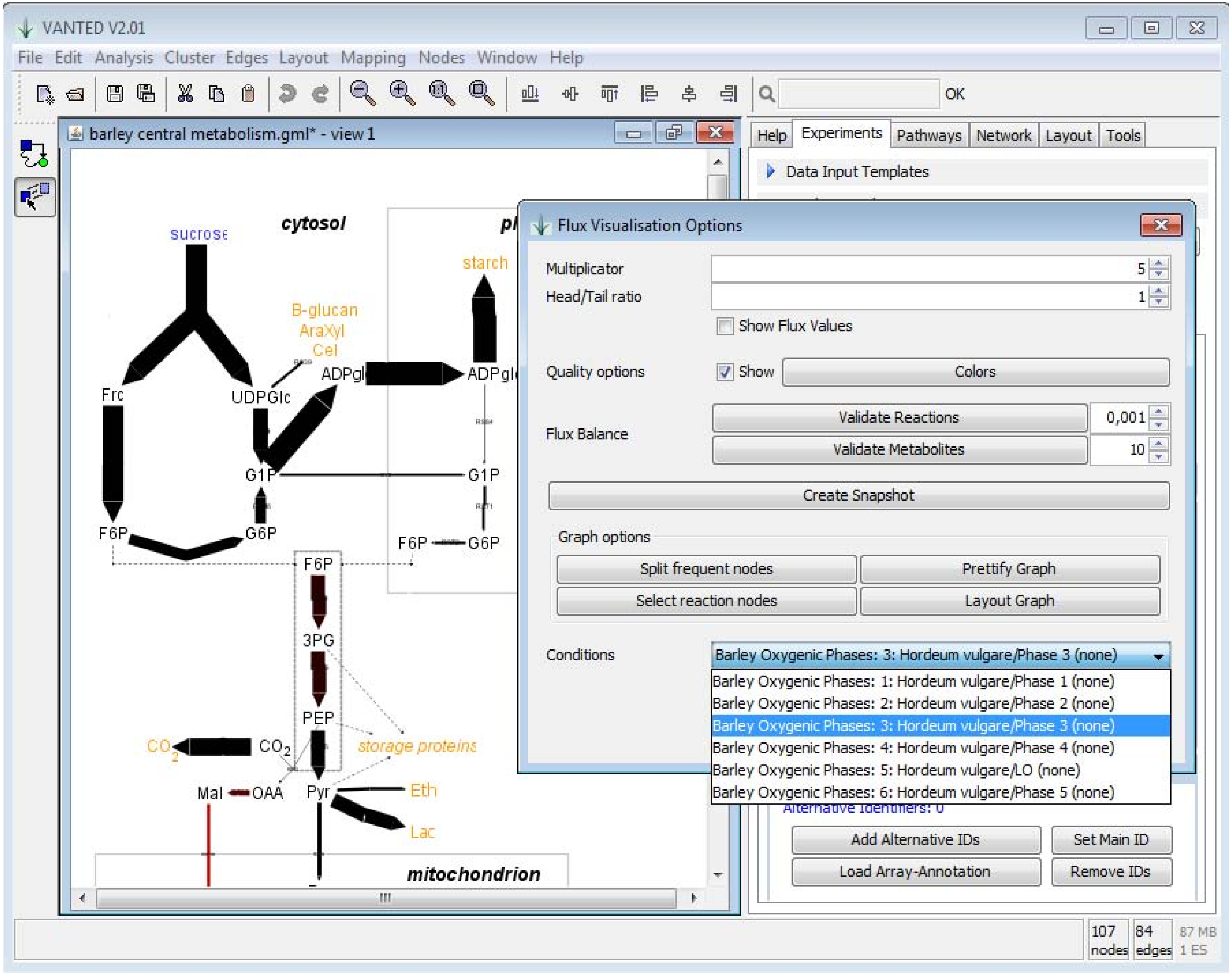

Figure 11: Switch between different conditions

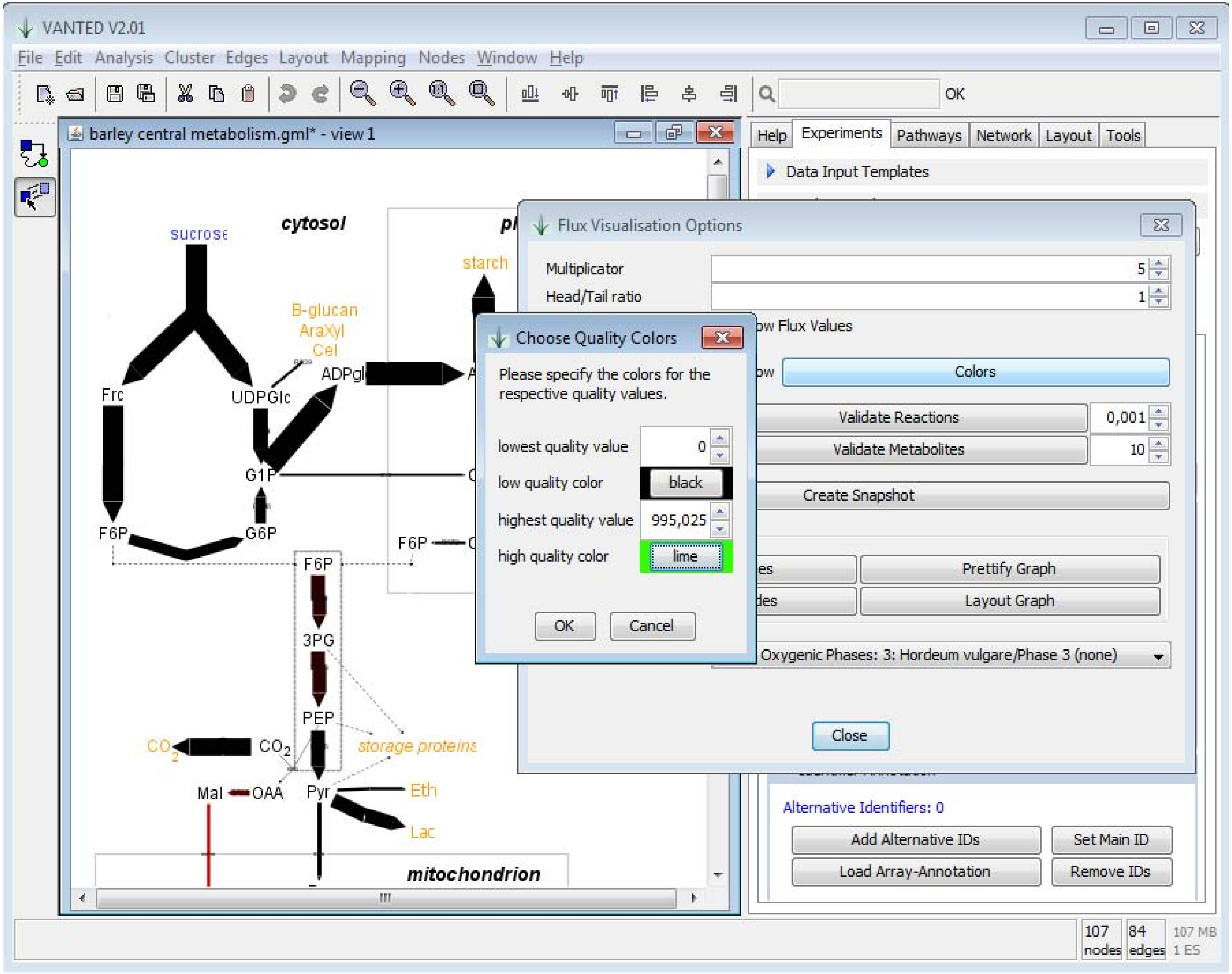

Figure 12: Adapt visualization of quality information

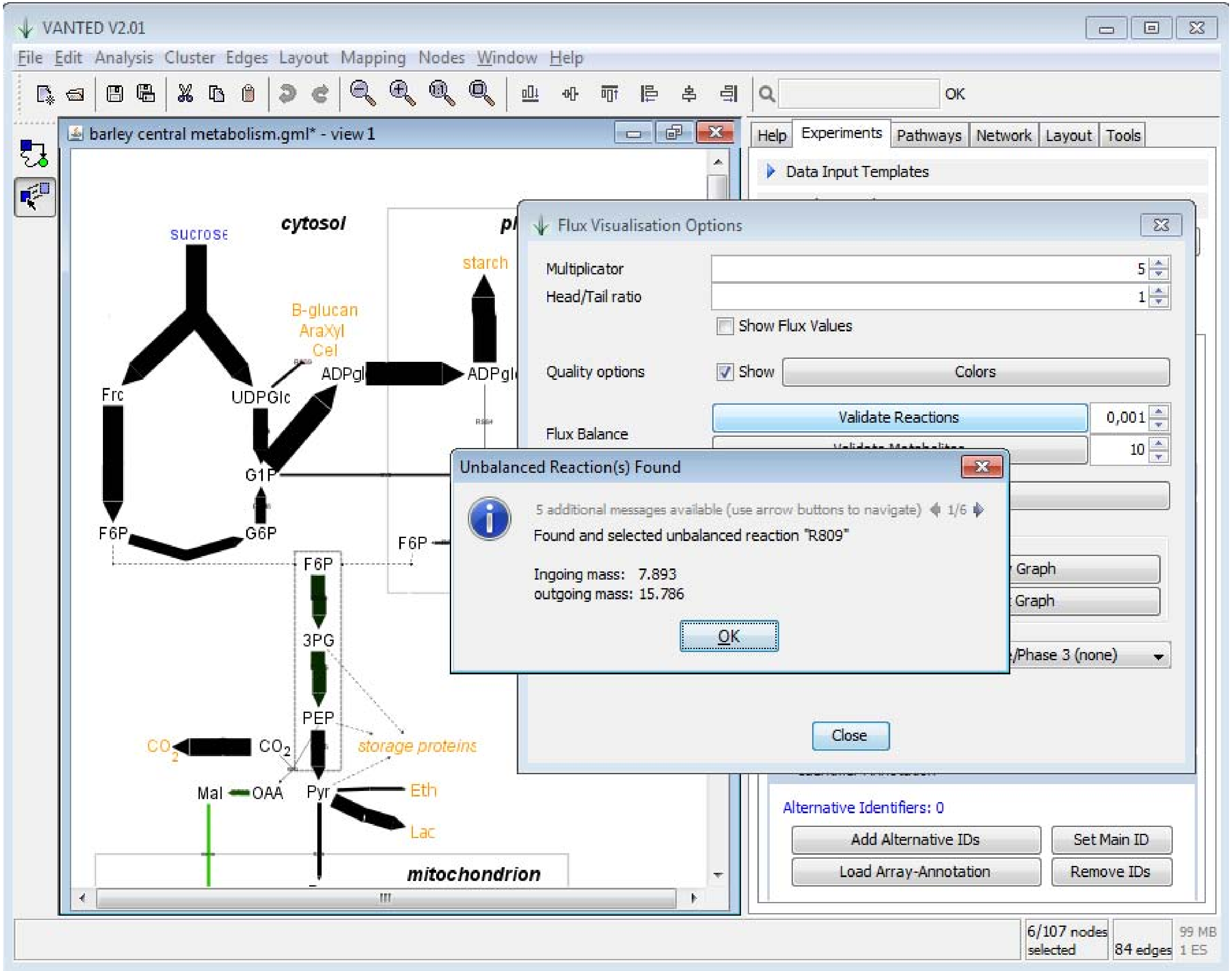

Figure 13: Validate flux balance

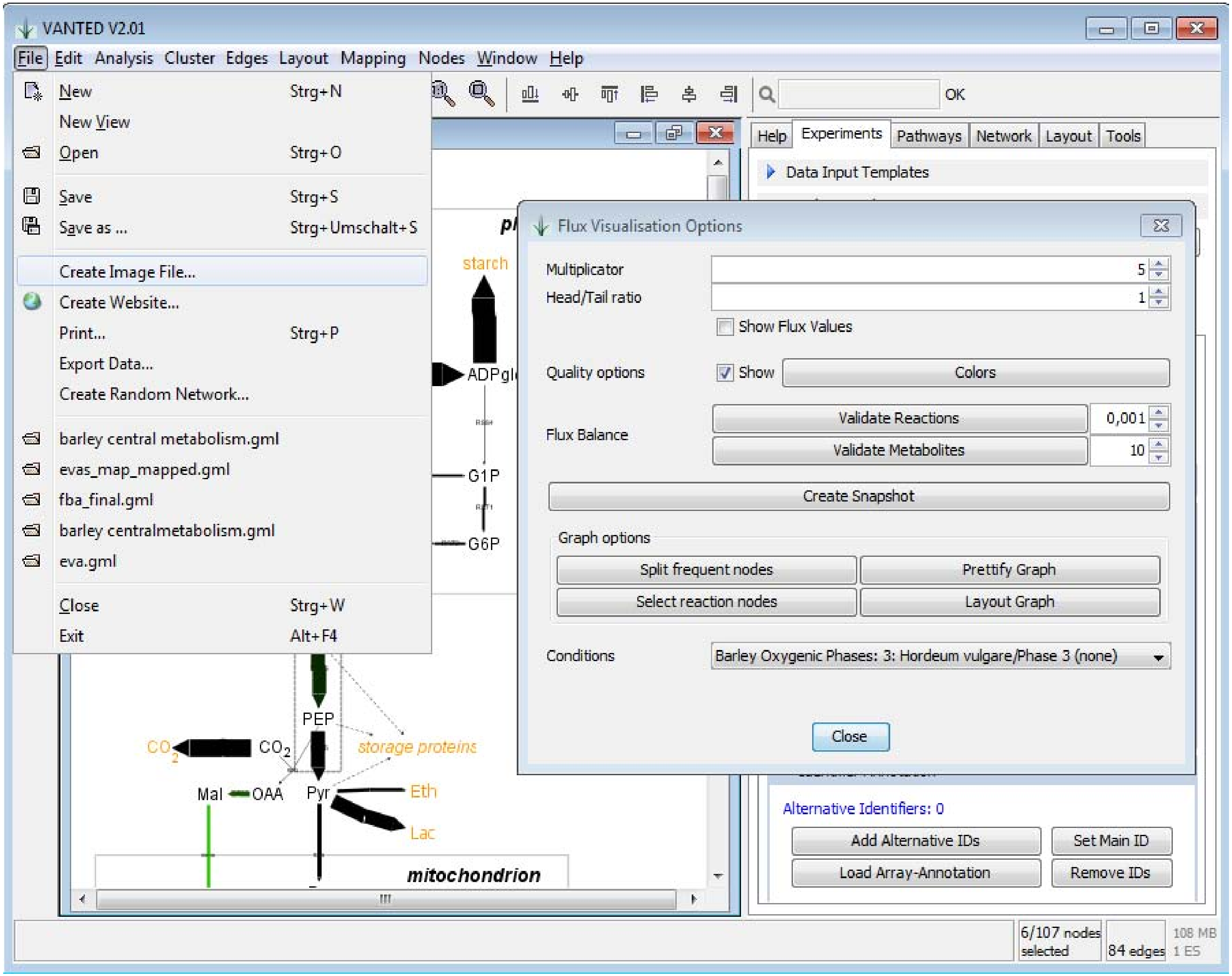

Figure 14: Export data, graphics or webpage

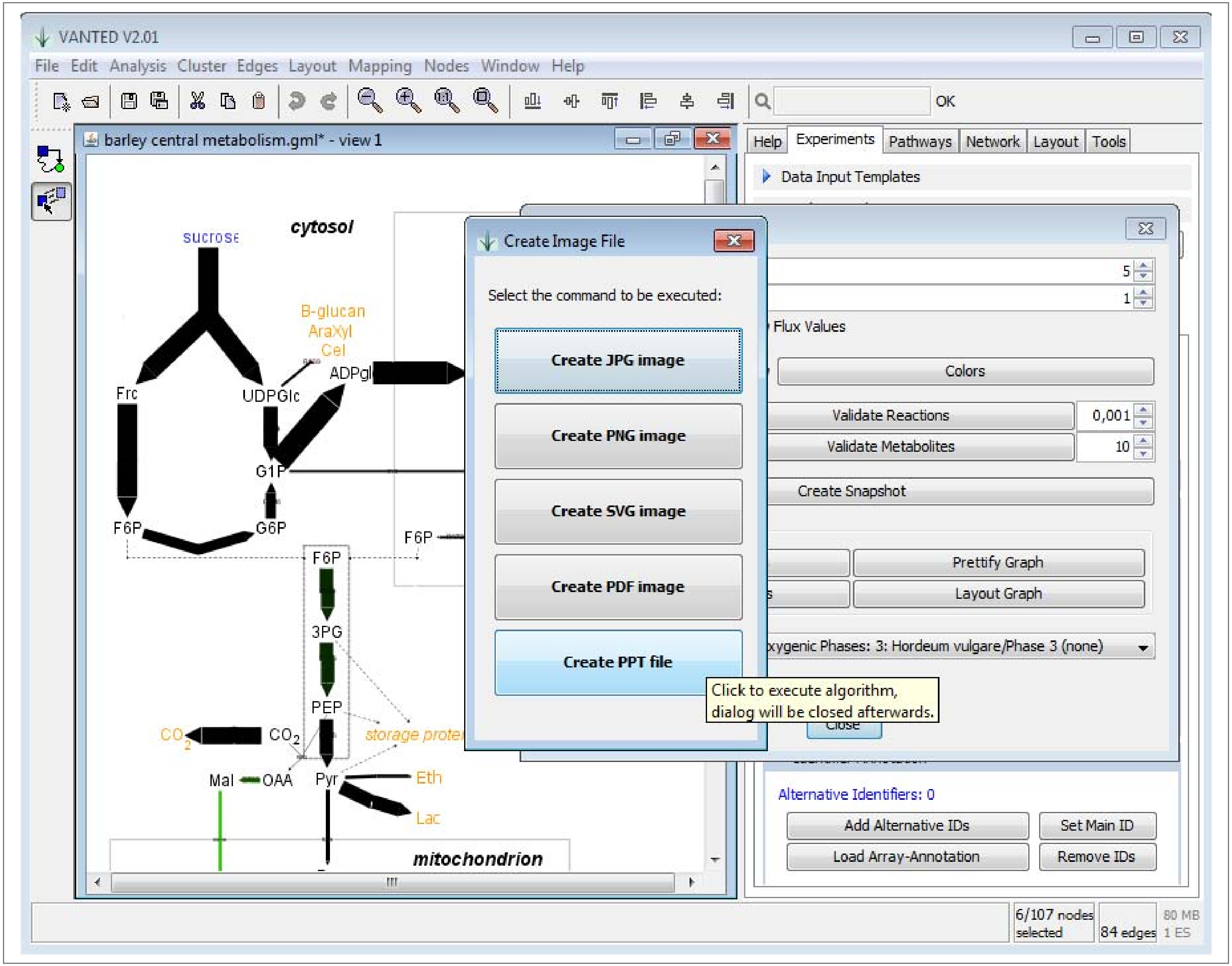

Figure 15: Export in different graphical formats

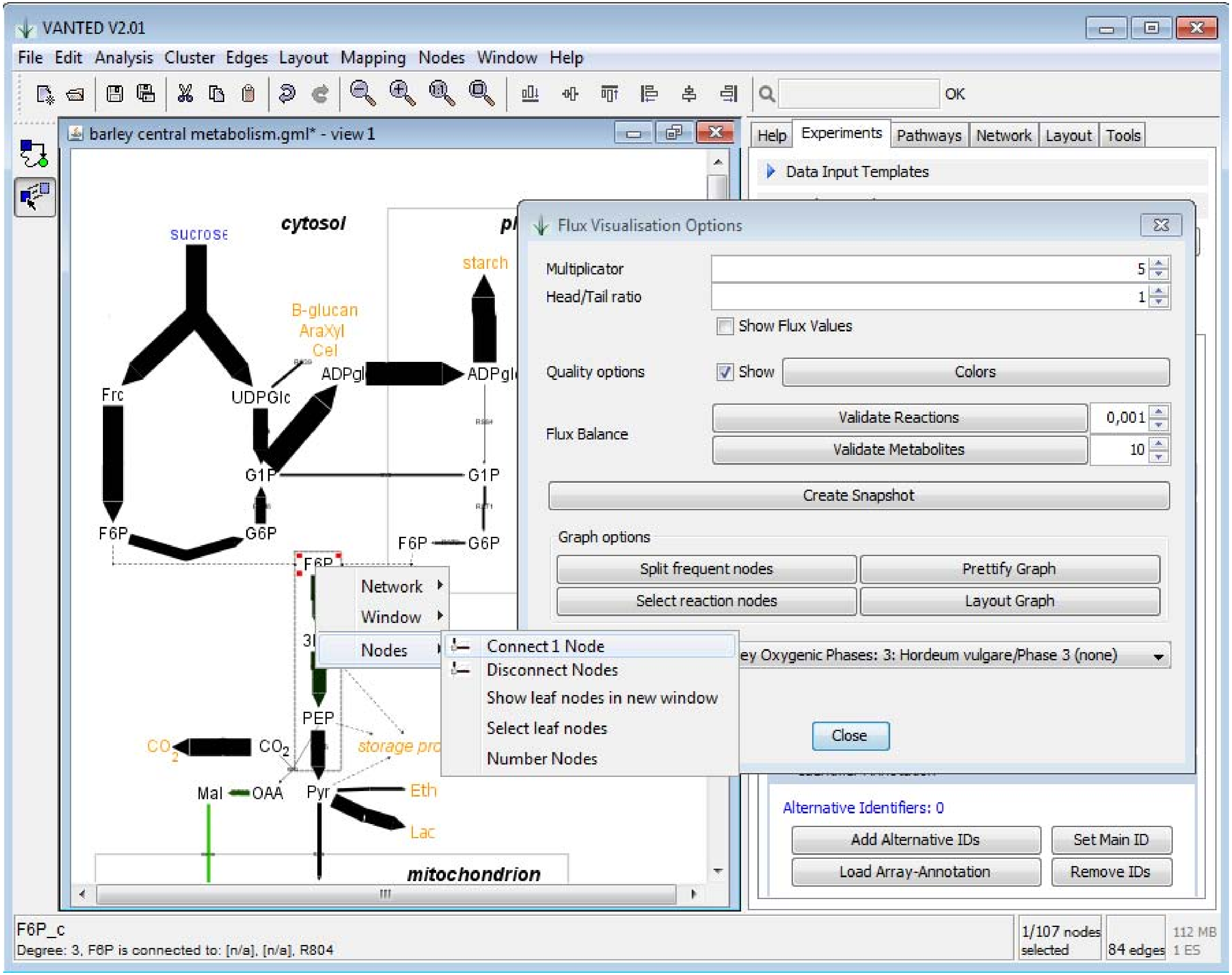

Figure 16: Connect metabolites with the same label

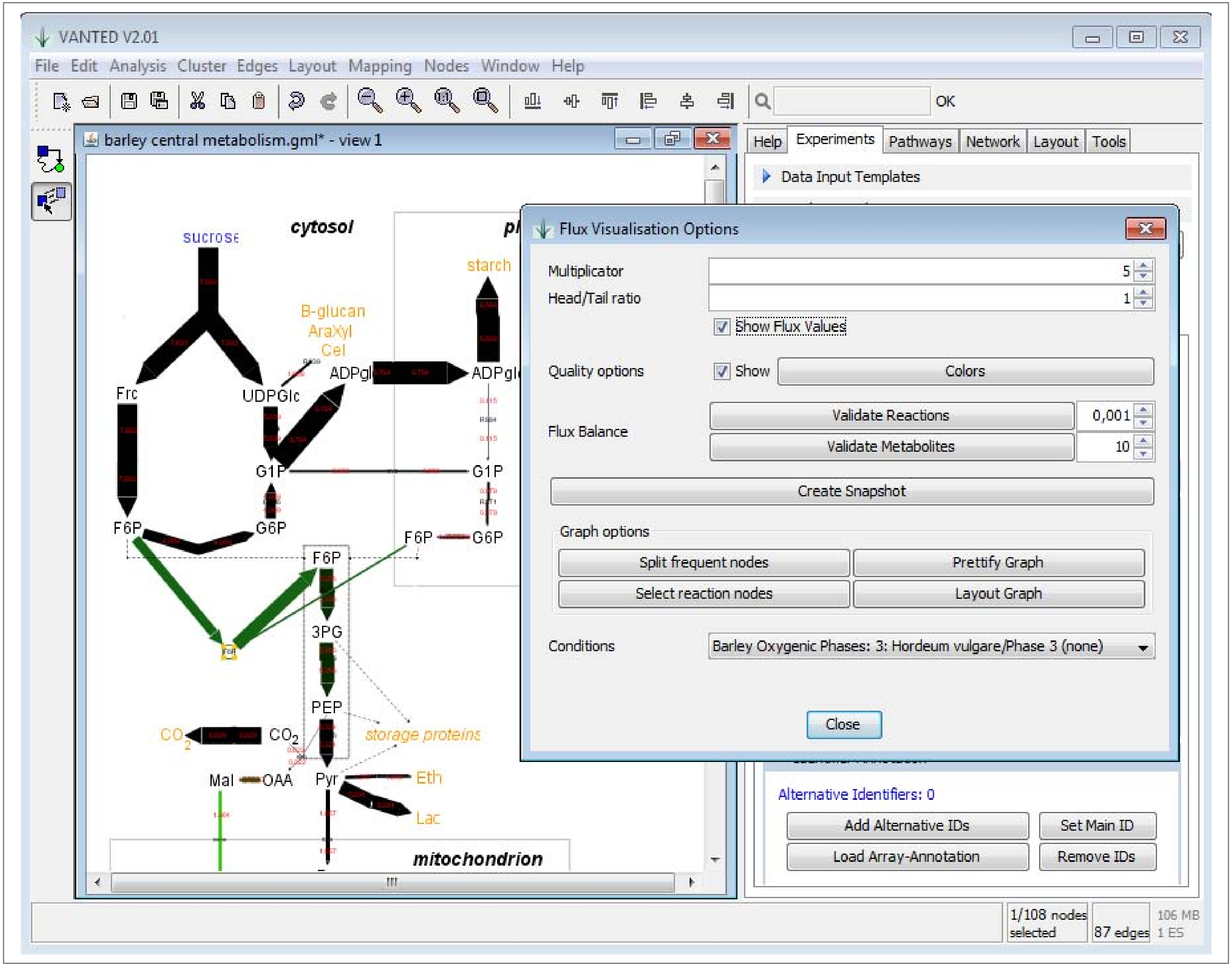

Figure 17: Show flux values on edges
